# Supplementary material for: Chitosan-Gelatin Films Cross-Linked with Dialdehyde Cellulose Nanocrystals as Potential Materials for Wound Dressings
Source: Int J Mol Sci. 2022 Aug 26;23(17):9700. doi: 10.3390/ijms23179700 (PMC9456065; doi:10.3390/ijms23179700)
Supplement: Supplementary file 1 [file ijms-23-09700-s001.zip › ijms-1872649-supplementary.pdf]

Supplementary Materials

Chitosan-Gelatin Films Cross-Linked with Dialdehyde Cellulose Nanocrystals as Potential Materials for Wound Dressings

Katarzyna Wegrzynowska-Drzymalska [1*], Dariusz T. Mlynarczyk [2], Dorota Chelminiak-Dudkiewicz [1], Halina Kaczmarek [1], Tomasz Goslinski [2], and Marta Ziegler-Borowska [1]

[1] Department of Biomedical Chemistry and Polymer Science, Faculty of Chemistry, Nicolaus Copernicus University in Torun, Gagarina 7, 87-100 Torun, Poland
[2] Chair and Department of Chemical Technology of Drugs, Poznan University of Medical Sciences, Grunwaldzka 6, 60-780 Poznan, Poland
* Correspondence: kasiawd@doktorant.umk.pl

Figure S1. ATR-FTIR spectra of chitosan-gelatin film... 3

Figure S2. AFM images in 2D and 3D scale of (A) chitosan-gelatin film and (B) chitosan-gelatin films cross-linked with (a) 5%DAMC, (b) 10%DAMC, (c) 15%DAMC, (d) 5%DNCL, (e) 10%DNCL, and (f) 15%DNCL. 4

Table S1. Amount of protein bound on the surface of biopolymers in mg of protein per 1 cm^2 of biopolymer film in the full range of incubation timescales.. 5

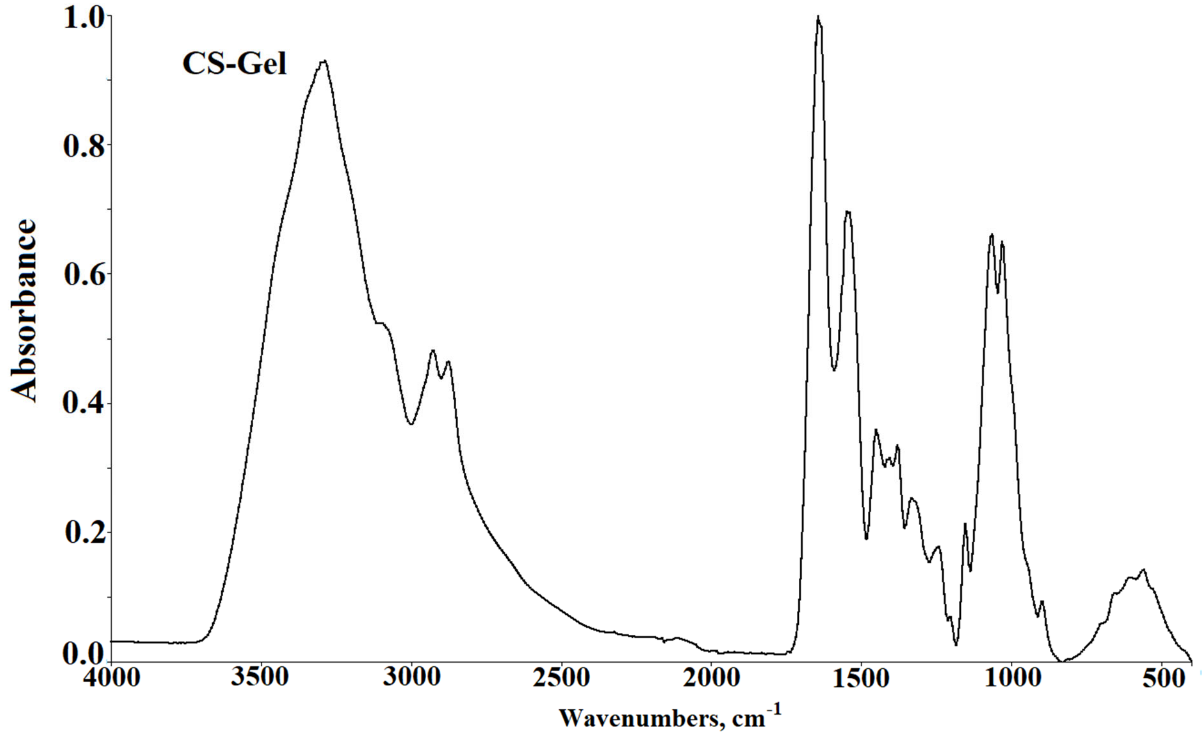

Figure S1. ATR-FTIR spectra of chitosan-gelatin film.

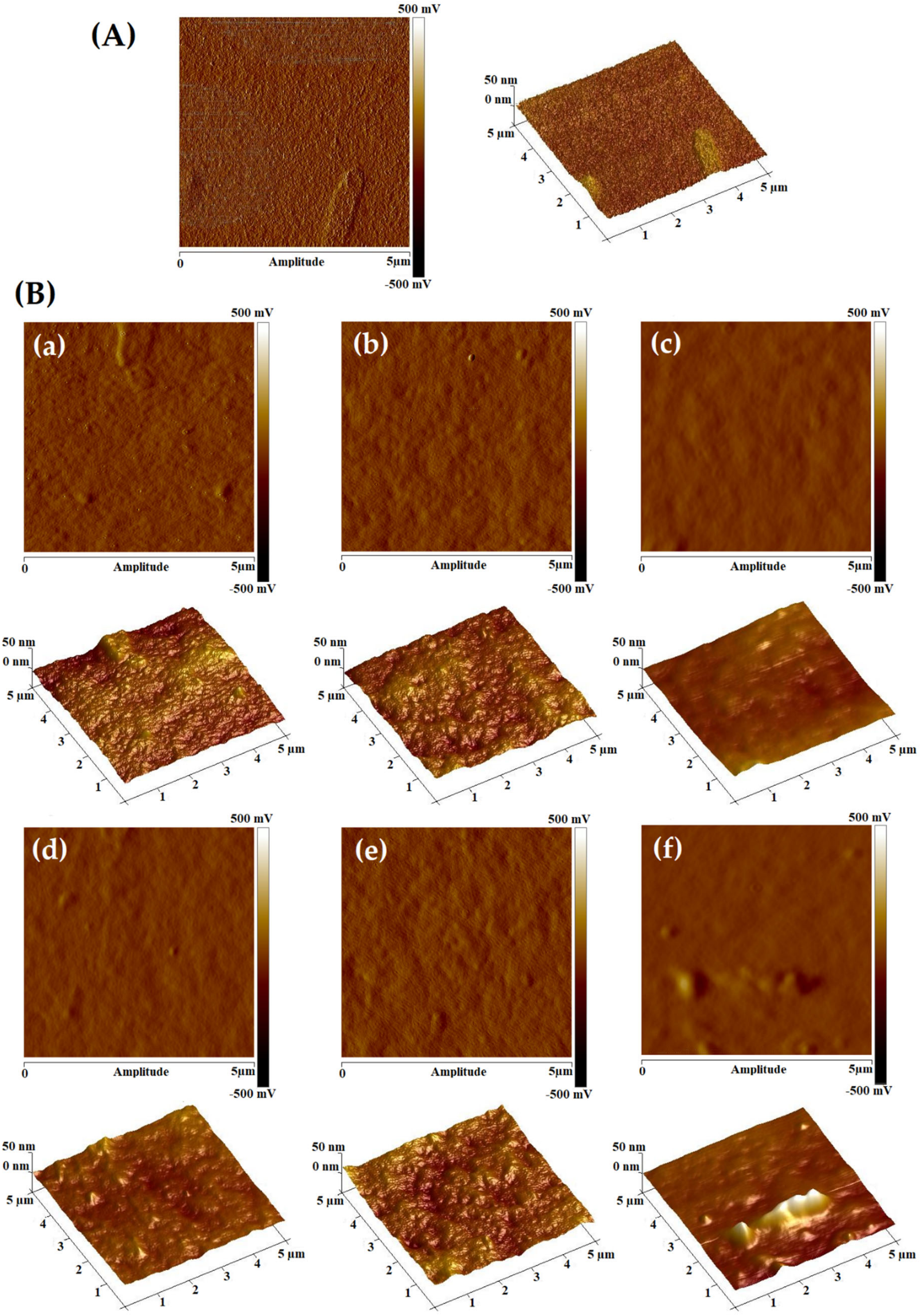

Figure S2. AFM images in 2D and 3D scale of (A) chitosan-gelatin film and (B) chitosan-gelatin films cross-linked with (a) 5%DAMC, (b) 10%DAMC, (c) 15%DAMC, (d) 5%DNCL, (e) 10%DNCL, and (f) 15%DNCL.

Table S1. Amount of protein bound on the surface of biopolymers in mg of protein per 1 cm^2 of biopolymer film in the full range of incubation timescales.

Sample	Incubation time						
	1h	2h	3h	4h	5h	6h	24h
	Amount of adsorbed human serum albumin (mg/cm^2)						
**CS-Gel**	0.024	0.027	0.029	0.031	0.030	0.033	0.036
**CS-Gel-5%DAMC**	0.033	0.037	0.028	0.030	0.035	0.038	0.041
**CS-Gel-10%DAMC**	0.064	0.065	0.049	0.049	0.047	0.048	0.052
**CS-Gel-15%DAMC**	0.074	0.090	0.094	0.062	0.059	0.058	0.054
**CS-Gel-5%DNCL**	0.058	0.051	0.070	0.033	0.030	0.034	0.036
**CS-Gel-10%DNCL**	0.053	0.045	0.067	0.030	0.030	0.028	0.027
**CS-Gel-15%DNCL**	0.069	0.056	0.075	0.043	0.039	0.040	0.043
